# Exploring Protein Bioconjugation: A Redox-Based Strategy for Tryptophan Targeting

**DOI:** 10.34133/research.0410

**Published:** 2024-07-04

**Authors:** Qian-Qian Yang, Shuai-Jiang Liu, Wei Huang, Cheng Peng, Bo Han

**Affiliations:** State Key Laboratory of Southwestern Chinese Medicine Resources, School of Pharmacy, Chengdu University of Traditional Chinese Medicine, Chengdu 611137, P. R. China.

## Abstract

Amino acid bioconjugation technology has emerged as a pivotal tool for linking small-molecule fragments with proteins, antibodies, and even cells. The study in *Nature* by Chang and Toste introduces a redox-based strategy for tryptophan bioconjugation, employing *N*-sulfonyloxaziridines as oxidative cyclization reagents, demonstrating high efficiency comparable to traditional click reactions. Meanwhile, this tool provides feasible methods for investigating the mechanisms underlying functional tryptophan-related biochemical processes, paving the way for protein function exploration, activity-based proteomics for functional amino acid identification and characterization, and even the design of covalent inhibitors.

In recent years, covalent inhibitors [1,2] and molecular chemical probes [3] designed based on nucleophilic amino acids have attracted widespread attention in the field of drug discovery. Compared to traditional non-covalent inhibitors, covalent inhibitors provide higher affinity by forming stable covalent bonds with target proteins. Additionally, covalent inhibitors help to circumvent some potential resistance mechanisms, especially when facing undruggable proteins [4–6] or kinases [7] (such as SHP2 and KRAS) that lack traditional binding pockets due to mutations. Covalent inhibitors can expand their therapeutic potential, offering possibilities for developing new therapeutic strategies, leading to an increasing number of small-molecule covalent inhibitors successfully entering the market [8]. The development of reactive “warheads” that rapidly and effectively covalently bind to specific amino acid residues is pivotal in the creation of covalent inhibitors. With the advancement of amino acid bioconjugation chemistry [9], numerous novel warheads have emerged in recent years, targeting a variety of amino acid residues including cysteine [10], methionine [11], tyrosine [12], etc. [13]. Unlike traditional approaches to targeting amino acid residues bearing highly nucleophilic groups, tryptophan residue, which contains an indole segment, shows weak nucleophilicity under physiological pH conditions, making it difficult to achieve covalent targeting using conventional acid–base catalysis methods. Currently, while a few methods offer strategies for covalently targeting tryptophan, achieving this under physiological conditions remains challenging [14,15], leading to the underdevelopment of bioconjugation reagents specific to tryptophan.

In a recent publication in *Nature*, Chang and Toste introduced a novel redox-based strategy for tryptophan bioconjugation [16], further advancing the selective coupling methods previously established for methionine [11]. This innovative approach draws inspiration from the indole oxidative cyclization biosynthesis, akin to the Communesin core [17]. Their approach utilized *N*-sulfonyloxaziridines (Davis reagents or Davis oxaziridines) [18], which are stable, aprotic, and neutral oxidizing agents. These reagents are capable of oxidizing a wide variety of nucleophiles with high regioselectivity and stereoselectivity [19], thereby presenting a novel strategy for tryptophan bioconjugation. This method, termed tryptophan chemical ligation by cyclization (Trp-CLiC), achieves selective and rapid functionalization of tryptophan at the levels of peptides, proteins, and proteomes, with a reaction rate comparable to traditional click chemistry reactions [20]. The authors demonstrated the broad application of this method in revealing functional tryptophan-related biochemical processes, including systematically identifying tryptophan residues involved in cation–π interactions, and deciphering these cation–π motifs could functionally regulate protein-mediated phase-separation processes, with disease-related mutations or post-translational modifications that disrupt these interactions leading to alterations in protein localization (Fig. 1).

**Fig. 1.**
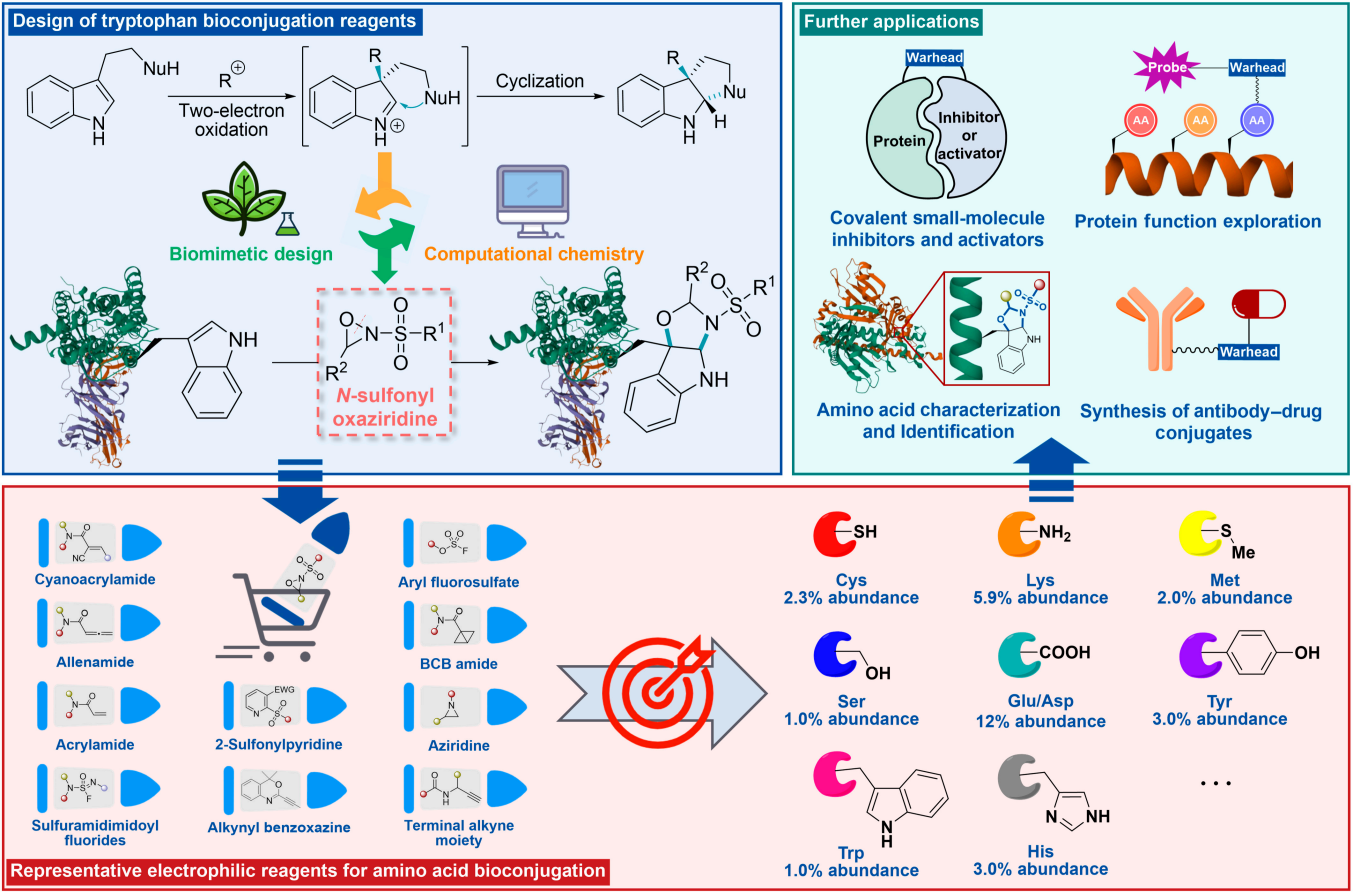
Utilizing *N*-sulfonyl oxaziridine as a specific covalent coupling reagent for tryptophan provides a novel strategic approach for the bioconjugation of nucleophilic amino acids, which offers highly efficient chemical "tools" for further applications related to bioconjugation.

Using biomimetic and computational chemistry screening, the authors found that *N*-sulfonyl oxaziridine showed high reaction efficiency, and after further optimization of substituents, they obtained the Ox-W18 with about 90% selectivity. Additionally, the Trp-CLiC method can be successfully applied to various known protein labeling, including site-specific antibody functionalization and monitoring protein unfolding in solution and cells. Furthermore, an activity-based protein proteomics (ABPP) approach based on Trp-CLiC has also been established for identifying new functional tryptophan sites in the cell proteome. Using this method, 906 tryptophan residue binding sites were identified on protein surfaces, mainly associated with undruggable proteins and highly enriched in various subcellular regions, especially in phase-separated subcellular compartments. Using the NPM1 protein as a model, further investigation into the relationship between functional tryptophan residues on the protein surface and cellular phase separation revealed that the 2 labeled tryptophan residues could engage in lysine–tryptophan cation–π interactions. Moreover, acylation modifications resulted in alterations in protein localization and phase separation, demonstrating the regulatory role of cation–π interactions in protein phase separation and localization.

Despite the relatively small proportion of tryptophan in the human body [10], tryptophan plays a key role in various proteins. The widespread application of the novel oxidative covalent coupling method in tryptophan-specific bioconjugation not only overcomes the challenges of targeting tryptophan for covalent coupling but also enriches the types of developed warheads, providing new avenues for the development of bioconjugation techniques targeting other amino acids. Firstly, the Trp-CLiC redox method serves as an effective tool for developing covalent small-molecule inhibitors and activators, while also aiding in the exploration of protein functionality, activity-based proteomics for functional amino acid identification and characterization, and the synthesis of antibody–drug conjugates. Secondly, by leveraging the structural characteristics of amino acid residues to develop various types of potential reaction pathways and integrating interdisciplinary knowledge from biochemistry, medicinal chemistry and computational chemistry, new insights are brought to the development of covalent inhibitors based on protein conjugation technology.

Moreover, compared to in vitro bioconjugation, the complexity of the biological environment often results in a lack of specificity in chemically based protein bioconjugation. Thus, covalent modifications of endogenous proteins in their native biological milieu are largely underdeveloped. The development of highly specific protein bioconjugation strategies, coupled with quantitative analysis using instruments of high sensitivity and accuracy, will be essential for future exploration and modification of endogenous proteins. The utilization of gene editing tools like the CRISPR/Cas9 system [21], which enables the expression of genetically engineered proteins as endogenous proteins, in conjunction with the aforementioned chemical methods, offers abundant opportunities for the chemical modification of endogenous proteins in live cells. Furthermore, with the burgeoning development of various deep learning models, systematic analysis of accumulated data may enable prediction of novel bioconjugation methods and covalent binding sites on proteins in the future, significantly expediting research progress and accelerating exploration in this field [22].
